# A Proposed Maneuver to Guide Transseptal Puncture Using Real-Time Three-Dimensional Transesophageal Echocardiography: Pilot Study

**DOI:** 10.1155/2015/174051

**Published:** 2015-05-27

**Authors:** Hani M. Mahmoud, Mohammed A. Al-Ghamdi, Abdullah E. Ghabashi, Ashraf M. Anwar

**Affiliations:** ^1^Adult Cardiology Department, Prince Sultan Cardiac Center, Al-Hassa, Saudi Arabia; ^2^Cardiology Department, Al-Azhar University, Cairo, Egypt

## Abstract

*Aim of Study*. To assess the feasibility of a new proposed maneuver “RATLe-90” using real-time three-dimensional transesophageal echocardiography (RT-3DTEE) for anatomically oriented visualization of the interatrial septum (IAS) in guiding the transseptal puncture TSP.* Methods*. The study included 20 patients (mean age, 60.2 ± 6.7 years; 60% males) who underwent TSP for different indications. RT-3DTEE was used to guide TSP. The proposed maneuver RATLe-90 (Rotate-Anticlockwise-Tilt-Left-90) was applied in all cases to have the anatomically oriented en face view of the IAS from the right atrial (RA) aspect. Having this anatomically oriented view, we guided the TSP catheter towards the proper puncture site according to the planned procedure*. Results*. Using the RATLe-90 maneuver, the anatomically oriented en face view of the IAS from the RA was obtained in all patients. We were able to guide the puncture catheter to the proper puncture site on the IAS. The 3D images obtained were clearly understood by both echocardiographers and interventionists. The RATLe-90 maneuver acquisition time was 19.9 ± 1.6 seconds. The time-to-tent was 64.8 ± 16.3 seconds. Less TEE probe manipulations were needed while guiding the TSP. *Conclusions*. Application of RT3D-TEE during TSP using RATLe-90 maneuver is feasible with shorter fluoroscopy time and minimizing TEE probe manipulations.

## 1. Introduction

Transseptal puncture (TSP) is a common step in various percutaneous structural cardiac interventions such as balloon mitral valvuloplasty [1], percutaneous mitral valve edge-to-edge repair [2], and percutaneous left atrial appendage (LAA) closure [3]. Two-dimensional transesophageal echocardiography (2D-TEE) is a widely used method for guidance of TSP [4]. However, 2D-TEE has the inherent disadvantage of being only two-dimensional which makes it very common for the echocardiographer to lose the view if the catheter went few millimeters out of the very thin imaging plane. With the advent of real-time three-dimensional TEE (RT3D-TEE) we are now capable of getting 3D anatomically oriented en face view for the interatrial septum (IAS) in real time [5].

Till now there are no standardized maneuvers or protocols to get the anatomically oriented en face view for the IAS during guiding the septal puncture.

The aim of this pilot study was to propose a simple and standardized maneuver to get the anatomically oriented en face view of the IAS from the right atrial (RA) perspective to use for guiding the TSP.

## 2. Methods

Using a commercially available ultrasound system (Philips iE33; Philips Medical Systems, Andover, MA) with a matrix-array 3D-TEE probe (X7-2 t; Philips Medical Systems), twenty patients were included in this study (mean age, 60.2 ± 6.7 years; 60% males). Ten of them underwent TSP during percutaneous mitral valve edge-to-edge repair and the other 10 patients underwent TSP during percutaneous LAA closure. The study protocol was approved by the local Institutional Review Board (IRB). Informed consents were taken from all patients involved in the study.

## 3. Transesophageal Echocardiography

All procedures were performed under general anesthesia. Before starting the catheterization, the TEE probe was introduced. The first position of the TEE probe into the mid-esophagus was guided by the *x*-plane view, with two perpendicular views on the interatrial septum (IAS), imaging optimally the fossa ovalis. Then the mid-esophageal bicaval view was obtained.

### 3.1. Imaging Protocol

**Figure 1:**
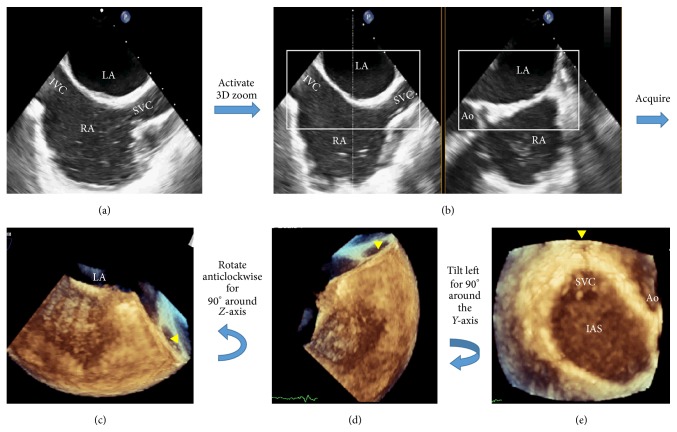
RATLe-90 Maneuver; starting by 2D-mid-esophageal 90° bicaval view (a), activate the 3D-zoom mode. The zoom box should be optimized to include the openings of the superior vena cava (SVC), inferior vena cava (IVC), and aortic root (Ao), with enough depth to include the whole interatrial septum (IAS) from both atrial perspectives excluding extra atrial tissues (b). Then, acquiring this volume will give us a truncated volume with the SVC (arrow head) pointing to the right of the screen, IVC pointing to the left, left atrial perspective of the IAS is up, and right atrial perspective is down (c). Then we will rotate anticlockwise this volume for 90° around the *z*-axis to have the SVC pointing superiorly (arrow head) and the IVC pointing inferiorly (d). Then we will Tilt-Left this volume for 90° around the *y*-axis to have the anatomically oriented en face view of the IAS from the right atrial (RA) perspective (e).

**Figure 2:**
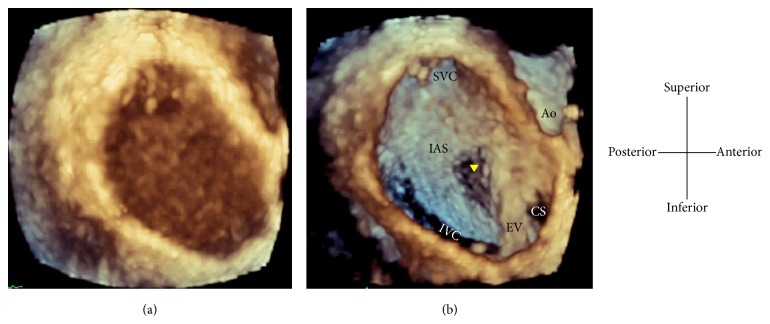
Reducing the gain will remove the blood signals and will allow clear identification of the right atrial structures, for example, SVC opening, IVC opening, Eustachian valve (EV), coronary sinus (CS) opening, and aortic root (Ao). Further gain reduction will cause dropout artifact in the thin area of the fossa ovalis (arrow head) that will help determining its location to guide septal puncture.

RATLe-90 “Rotate-Anticlockwise-Tilt Left for 90 degrees” maneuver:

1. Image acquisition: Starting from the two-dimensional transesophageal (2D-TEE) mid-esophageal bicaval view at around 90 degrees, the 3D zoom mode was activated and the zoom box was optimized in the lateral plane to include the ostia both of vena cava and in the elevation plane to include the aortic valve as well as the posterior atrial wall. With optimizing the depth of both planes to include the whole septum without the rest of the right atrial wall beyond the vena caval openings. After acquiring the volume, a real-time volume for the IAS was obtained with the superior vena cava (SVC) opening pointing to the right of the screen.
2. Maneuver: by rotating anticlockwise this volume for 90 degrees, the SVC opening is pointing superiorly. Then by Tilting-Left this volume for 90 degrees, the anatomically oriented en face view of the IAS from the RA perspective was clearly visualized (Figure 1) (Video 1 in Supplementary Material available online at http://dx.doi.org/10.1155/2015/174051).
3. By reducing the total gain, the blood intensities that filled the RA were eliminated in order to show the anatomical structures inside the RA. Gain adjustment to a certain level enabled us to create a dropout artifact in the thin area of the septum (fossa ovalis) in order to identify its location (Figure 2) (Video 1).

### 3.2. Guiding Transseptal Puncture (TSP)

**Figure 3:**
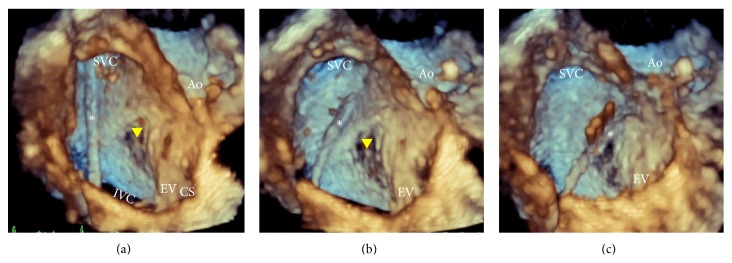
RT3DTEE anatomically oriented view of the IAS from the RA perspective during guidance of transseptal puncture. The puncture catheter (asterisk) was introduced through the IVC into the SVC (a). Then the catheter (asterisk) was pulled inferiorly and anteriorly towards the thin part of the IAS (arrow head) “Fossa” (b and c).

The interventionist passed the catheter from the femoral vein through the IVC into the SVC. Direct real-time visualization of the needle tip and the fossa using 3D-TEE was obtained to navigate the catheter till it reached the fossa ovalis for tenting (Figure 3).

#### 3.2.1. Time-to-Tent

We calculated the time from the first motion of the TSP catheter from the SVC downwards till the septal tenting at the required position.

Retrospectively, we calculated the “time-to-tent” in a control group of twenty patients who underwent TSP for its various indications in our center using fluoroscopy as well as 2D-TEE for guidance.

## 4. Results

We could get the en face anatomically oriented view of the IAS from the RA perspective in all of the 20 patients included in this study through using the RATLe-90 maneuver within a mean time of 19.5 ± 1.6 seconds starting from a standard mid-esophageal bicaval view.

Using this maneuver, probe manipulations were minimized regardless of the motion of the equipment used by interventionist (needles, sheaths, and catheters). Compared to 2D-TEE, the acquisition time was reduced.

In patients who underwent TSP for LAA closure, the location of fossa ovalis was clearly visualized and the orientation of the whole IAS helped the interventionist to direct the needle and catheter to the proper TSP site, that is, posteroinferior portion of the fossa ovalis.

In patients who underwent MV repair, the location of fossa ovalis was clearly visualized and the orientation of the whole IAS helped the interventionist to direct the needle and catheter to the proper TSP site, that is, posterosuperior portion of the fossa ovalis.

Better communication between the echocardiographer and the interventionist was achieved in terms of directions of motions of the catheter as both of them will agree that upward motion of the catheter in the fluoroscopy as well as in the echocardiography screens means superior and downward motion means inferior and so forth. Also, having the catheter as well as the IAS and fossa in the same real-time 3D view while navigating with the catheter over the IAS is a unique opportunity that is more comfortable for the echocardiographer that does not need to manipulate the TEE probe with every motion of the needle over the septum to keep it in view.

Time-to-tent for the study group was (mean, 64.8 ± 16.3 seconds), while for the control group was (mean, 139.9 ± 29.3 seconds); *P* value was less than 0.0001.

In all patients, TSP was not complicated by pericardial effusion or cardiac perforation due to improper puncture of RA wall.

## 5. Discussion

2D-TEE had been used widely as adjunctive with fluoroscopy to guide TSP for its various indications [1–3]. But, thanks to the technological development, now we have the fully sampled matrix array 3D-TEE transducers that made it possible to have a wider sector live/real-time images for the beating heart in three dimensions [5].

It is also worthy to mention that if the 2D image quality is poor one should not try using 3D imaging as it is going to be worse because of the lower spatial and temporal resolution regardless of the used acquisition mode. It is also sometimes difficult to get a proper mid-esophageal bicaval 2D-TEE view with the septum in a more-or-less flat position which will badly affect the quality of the acquired 3D-zoomed view that may not show the inferior portion of the IAS properly.

Although tachycardia is usually problematic during 3D-TEE due to the relatively lower frame rate and temporal resolution of this modality, fortunately, the IAS is a relatively static structure that is not too much affected by the lower frame rate and temporal resolution.

The anatomically oriented en face view for the IAS from the RA perspective was described before for the purpose of delineating the anatomy of atrial septal defects [6]. It was also reported by us to be used for guiding coronary sinus CS cannulation in percutaneous mitral annuloplasty using CS ring [7].

In our study we measured the time-to-tent instead of time-to-puncture to avoid bias related to technical issues like slipping of the catheter over the septum due to improper catheter on needle motion or delayed puncture due to a stiff abnormal septum, believing that the role of TEE guidance is only to guide the TSP catheter to the proper tenting position.

To the best of our knowledge, no previous study reported the use of RT3D-TEE driven anatomically oriented view of the IAS for guidance of the transseptal puncture (TSP). The current study showed the value of RT3D-TEE application during TSP during LAA closure and MV repair procedures. Using a step wise maneuver (RATLe-90) helped in minimizing the time needed to visualize the IAS in multiple echocardiographic views. By this maneuver, the anatomically oriented view of IAS during TSP was obtained and thus created a common language between the echocardiographer and the interventionist. This was reflected in reducing the time needed for TSP compared to previous studies which relied on 2D-TEE.

The findings of this pilot study encouraged us to enroll all patients who will undergo TSP in various interventional procedures and a large study is planned.

## 6. Conclusion

Application of RT3D-TEE during TSP using RATLe-90 is feasible with better acquisition time, shorter fluoroscopy time, and minimizing TSP complications.

## Supplementary Material

Video legend: RATLe-90 Maneuver; Starting from the 2D-mid-esophageal 90 ͦ bi-caval view, activate the 3D-zoom mode. The zoom box should be optimized to include the openings of the superior vena cava (SVC), inferior vena cava (IVC) & Aortic root (Ao), with enough depth to include the whole inter-atrial septum (IAS) from both atrial perspectives excluding extra atrial tissues. Then, acquiring this volume will give us a truncated volume with the SVC pointing to the right of the screen (arrow-head), IVC pointing to the left. Then Rotate-Anticlockwise this volume for 90ͦ to have the SVC pointing superiorly (arrow-head) & the IVC pointing inferiorly. Then Tilt-Left this volume for 90ͦ to have the anatomically oriented enface view of the IAS from the right atrial (RA) perspective. Reducing the gain will remove the blood signals and allow clear identification of the SVC opening, IVC opening, Eustachian valve (EV), Coronary Sinus opening (CS) & will cause dropout artifact in the thin area of the fossa ovalis (arrow-head) that will help determining its location to guide septal puncture.
